# Adaptability and Stability Study of Selected Sweet Sorghum Genotypes for Ethanol Production under Different Environments Using AMMI Analysis and GGE Biplots

**DOI:** 10.1155/2016/4060857

**Published:** 2016-09-29

**Authors:** Justice Kipkorir Rono, Erick Kimutai Cheruiyot, Jacktone Odongo Othira, Virginia Wanjiku Njuguna, Joseph Kinyoro Macharia, James Owuoche, Moses Oyier, Alex Machio Kange

**Affiliations:** ^1^Department of Biochemistry and Molecular Biology, Egerton University, P.O. Box 536-20115, Egerton, Kenya; ^2^Department of Crops, Horticulture and Soil, Egerton University, P.O. Box 536-20115, Egerton, Kenya

## Abstract

The genotype and environment interaction influences the selection criteria of sorghum (*Sorghum bicolor*) genotypes. Eight sweet sorghum genotypes were evaluated at five different locations in two growing seasons of 2014. The aim was to determine the interaction between genotype and environment on cane, juice, and ethanol yield and to identify best genotypes for bioethanol production in Kenya. The experiments were conducted in a randomized complete block design replicated three times. Sorghum canes were harvested at hard dough stage of grain development and passed through rollers to obtain juice that was then fermented to obtain ethanol. Cane, juice, and ethanol yield was analyzed using the additive main effect and multiplication interaction model (AMMI) and genotype plus genotype by environment (GGE) biplot. The combined analysis of variance of cane and juice yield of sorghum genotypes showed that sweet sorghum genotypes were significantly (*P* < 0.05) affected by environments (E), genotypes (G) and genotype by environment interaction (GEI). GGE biplot showed high yielding genotypes EUSS10, ACFC003/12, SS14, and EUSS11 for cane yield; EUSS10, EUSS11, and SS14 for juice yield; and EUSS10, SS04, SS14, and ACFC003/12 for ethanol yield. Genotype SS14 showed high general adaptability for cane, juice, and ethanol yield.

## 1. Introduction

Sweet sorghum is gaining popularity for ethanol production due to its high sugar level in their stem juice. It is widely grown for food, feed, and fuel in semiarid tropics of Asia, Africa, America, and Australia [1] due to its drought tolerance. Drought is regarded as important abiotic stress causing yield instability and food insecurity [2]. Drought can be mitigated through irrigation as one of the available options; however, developing countries find it challenging due to huge capital investment. The introduction of drought-tolerant crops such as sorghum in the arid and semiarid lands (ASALs) remains the most desirable alternative. Sweet sorghum accumulates high amount of fermentable sugars in the stem. Uses of sweet sorghum include brewing for both industrial and local products and baking and home consumption as food. Sorghum is a multipurpose crop which can be adopted in semiarid parts of the country to help in the eradication of poverty through the supply of grain for food and sale of the stem to distilleries for ethanol production.

Studies of adaptability and stability provide information about the behaviour of each genotype under different environmental conditions. The phenotypic performance of each genotype is influenced by abiotic and biotic factors; some genotypes may perform well in one environment but fail in several others [3]. These factors include rainfall, temperature, soil fertility, light, pests, and diseases that vary across locations and significantly influence yield ability of crop varieties. These factors make it difficult to establish the superiority of cultivar across diverse environments [4]. A major drawback in the selection of genotypes with high yielding capacity in different environments is genotype by environment interaction. New genotypes must be stable for yields and should be stable across environments or suited to target regions [5]. Yield is controlled by the complex polygenic system and strongly varies depending on environmental conditions [6]. Stability analysis is an important step in developing cultivars for a wide range of environments or for a specific location [7]. Genotype by environment interaction has to be studied for yields, which are cane, juice, and ethanol in our case as they are considered the most important economic traits [8].

The genotype, environment, and the genotype by environment interactions impact crop performance. Genotype by environment interaction (GEI) complicates breeding, testing, and selection of superior genotypes [9]. The GEI changes the rankings of genotypes in various environments; an increase in GEI diminishes the correlation between genotypic and phenotypic qualities making it hard to distinguish superior genotype across environments [10]. The performance stability concept is therefore important in analyzing GEI in order to recommend genotypes to test environments. Additive main effects and multiplicative interactions (AMMI) analysis is used to determine stability of genotypes across locations using the principal component axis (PCA) scores and AMMI stability values (ASV) while genotype plus genotype by environment (GGE) analysis is effective method which is based on principal component analysis to fully explore multienvironment trials [11]. Average environment coordinates (AEC) of GGE biplot separates entries with below-average means from those with above-average means [12]. Stability of various crops has been studied by applying AMMI and GGE biplots successfully in soybean (*Glycine max* L. Meril) [13], sweet potatoes (*Ipomoea batatas*) [8], pepper (*Capsicum annuum*) [6], finger millet (*Eleusine coracana*) [14], wheat (*Triticum aestivum*) [15], grain sorghum [16], and rice (*Oryza sativa*) [17]. GGE and AMMI analysis were applied to determine stability and adaptability of eight sorghum genotypes grown in five different ecological zones.

## 2. Materials and Methods

### 2.1. Site Description

Sweet sorghum field experiments were carried out in Kisumu, Siaya, and Busia Counties of Kenya. The specific sites were Sinyanya (00°06′68.5′′S; 034°08′66.0′′E) at 1168 m above sea level (ASL), Masumbi (00°01′73.0′′N; 034°21′87.4′′E) at 1370 m ASL both in Siaya County, Mundika (00°24′56.6′′S; 034°07′93.1′′E) at 1222 m ASL in Busia, Nyahera (00°0.02′52.78′′S; 034°39′03.59′′E) at 1387 m ASL, and Sagam (00°03′20.86′′N; 034°32′31.06′′E) at 1216 m ASL both in Kisumu County.

**Figure 1:**
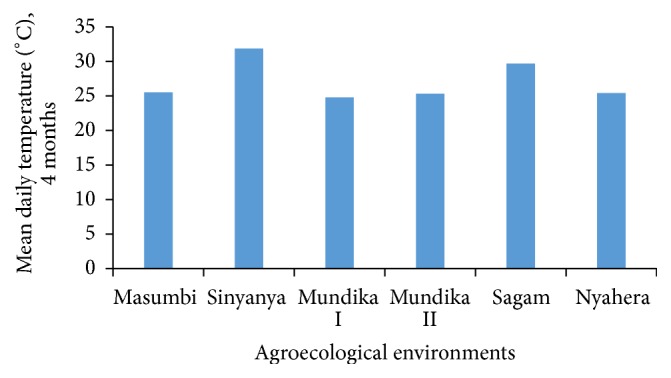
Mean daily temperature during sorghum growing period.

**Figure 2:**
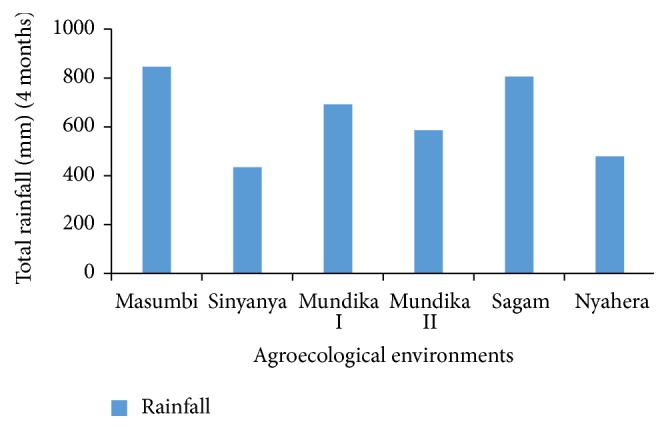
Cumulative rainfall during sorghum growing period.

All sites fell within the same agroecological zone, lower midland (LM); the difference in yield was due to difference in sub agroecological zones as depicted in Table 1. The environments in lower midland zones 1, 2, and 3 receive annual average rainfall of 1800–2000, 1550–1800, and 1200–1420 mm, respectively [18]. In general, the soil in these areas was sandy clay loam, acidic, or slightly acidic (pH = 4.4–6.0) and was poor in nitrogen and phosphorous. Sinyanya was characterized by high mean maximum temperature (Figure 1) and lower precipitation (Figure 2). The thermal zone 1 (LM1) in Kenya records mean daily temperature and altitude range of 22.2–21.0°C and 1200–1440 m ASL, respectively. The mean daily temperature and altitude range are 22.2–21.4°C and 1200–1350 m ASL, respectively, for LM2 and 22.7–21.0°C and 140–1500 m ASL for LM3 [19]. The lower midland zones 1, 2, and 3 are regarded as sugarcane, marginal sugarcane, and cotton zones, respectively [20].

### 2.2. Experimental Design

Eight sweet sorghum genotypes were grown in a randomized complete block design (RCBD). The genotypes were EUSS10, EUSS11, and EUSS17 as candidates with the controls being ACFC003/21, SS04, SS14, SS21, and SS17. Sowing was done on 18th March in Sinyanya and Masumbi and 19th March 2014 in Mundika for first season. Sowing in the second season was done on 13 September 2014 for both Mundika and Sagam while Nyahera was planted on 24 September 2014. Genotypes were sown in plots measuring 4 × 2.5 m in RCBD with three replications. Each plot consisted of four rows of sorghum at a spacing of 60 cm by drill and the blocks were separated by 1.5 m path. Triple superphosphate fertilizer was applied uniformly to all plots at a rate of 17.2 kg per ha before sowing. Control of weeds was done manually using hoes, three weeks after seedling emergence, and sorghum were thinned to a spacing of 10 cm within the row. And then, calcium ammonium nitrate (25% N) was top-dressed at the rate of 20 kg N/ha. Birds guarding was effected soon after the panicles formed to prevent grains damage.

### 2.3. Data Collection

Emergence was observed in all plots two weeks after planting, and stand counts were determined for all sorghum experimental units. Days to 50% heading were determined by calculating a number of days from sowing to when 50% of the sorghum heads in each plot had formed panicle. At dough stage of grain, plant height of each genotype was determined, and their panicles were harvested. Three randomly selected plants from each genotype in all replicates were used for recording plant height. Plant height was measured from base of the stem to tip of panicle and data averaged across three plants.

Harvesting took place on 9 July 2014 at the three sites: Masumbi, Mundika, and Sinyanya for the first season while Sagam and Mundika were harvested on 12 December 2014 and Nyahera on 29 December 2014 for the second season. Eight different genotypes were harvested at hard dough stage of grain taking plants in two inner rows of each plot. The leaves were stripped off by hand from harvested stalk and panicles removed using secateurs. The harvested stalks were weighed with a weighing balance to get fresh cane weight and then transported to the laboratory for juice extraction. Juice from the stalk was extracted in one roller crusher (Fuan Liyuan, China, type YC 80B-4) and strained through a sieve into a juice container. The volume of juice was measured, and Brix (%) was taken using hand refractometer.

### 2.4. Ethanol Analysis

Juice was sampled from each plot taking 100 mL for fermentation. Yeast (1.5%),* Saccharomyces cerevisiae*, was added to juice and fermentation process carried out at 35°C for four days and then distilled to obtain ethanol whose volume was determined. Refractometer (RFM 3330 code 25-330, Bellinghant Stanley limited) was used to determine the concentration of ethanol. The refractive index of distillate was taken and then compared with that of a standard curve. Absolute ethanol was mixed with distilled water to give the concentration of 0, 5, 10, 15, 20, 25, and 30% ethanol whose refractive index was taken to obtain standard curve showing the relationship between the refractive index and percent of ethanol in the distillate.

### 2.5. Statistical Analysis

Statistical computations were carried out using Genstat software version 15.1 (VSN International limited, 2012) for AMMI and GGE biplot analysis. The graphic representation of genotypes and environments by AMMI analysis results from a model of main additive effects and multiplicative interaction [21]. This model is expressed mathematically by

(1) $$\begin{array}{c} Y_{GEr}=\mu +\alpha_{G}+\beta_{E}+\underset{n}{\sum }\lambda_{n}\gamma_{Gn}\delta_{En}+\rho_{GE}+\varepsilon_{GEr}, \end{array}$$

where *Y*_GEr_ is mean yield of genotype G in the environment E for replication r; *μ* is grand mean; *α*_G_ is deviation of the genotype G from the grand mean; *β*_E_ is deviation of the environment E from the grand mean; *λ*_*n*_ is singular value for the interaction principal component axis (IPCA) *n*; *γ*_G*n*_ is the PCA score of a genotype for PCA axis *n*; *δ*_E*n*_ is the environmental PCA score for PCA axis *n*; *ρ*_GE_ is AMMI residual; and *ε*_GE_ is the error term when the experiment is replicated.

AMMI stability value (ASV) was calculated by

(2) $$\begin{array}{c} ASV=\sqrt{{\left[\frac{{SS}_{IPCA1}}{{SS}_{IPCA2}}\left(IPCA1score\right)\right]}^{2}+{\left(IPCA2score\right)}^{2}}. \end{array}$$

AMMI analysis was used to determine the stability of genotypes across locations using principal component axis (PCA) scores and ASV. Genotypes having the least ASV were considered as widely adapted genotypes. Similarly, IPCA2 scores close to zero revealed more stable genotypes, while large values indicated more responsive and less stable genotypes. GGE biplot analysis was used to visualize the relationship between testers and entries and to determine “which-won-where” portion. GGE biplot also reveals stability of genotypes; genotypes located near the biplot origin are considered as widely adapted genotypes while genotypes located far are considered as being specifically adapted.

## 3. Results and Discussion

### 3.1. Effect of Environment on Maturity, Plant Height, and Biomass of Selected Sweet Sorghum

The effects of genotype were significant on days to 50% heading, with SS21 being early maturing genotype across environments (Table 2). Genotypes SS14, EUSS10, and EUSS11 took long to mature across environments. The time difference between early and late maturing genotypes was more than two weeks across environments except in Masumbi and Mundika (2nd season). Generally, genotypes matured earlier during the second season compared to the first season. From the study, it was observed that the least number of days to reach 50% heading was about eight and a half weeks (61 days).

Plant height differed among sweet sorghum genotypes and across locations. The tallest and shortest plant height were recorded by genotypes in Sagam and Sinyanya, respectively (Table 3). Genotype SS21 was the shortest during the first and the second season. SS04, EUSS10, and ACFC003/12 grew taller consistently across environments. In Nyahera, the results indicate plant height was similar for all genotypes ranging from 162 to 181 cm except for ACFC003/12 (189 cm), though the difference among ACFC003/12, EUSS10, SS21, SS04, and SS17 was not significant. Similarly, in Masumbi, all genotypes were similar in height except for SS21 that was recorded to be 66 cm shorter than EUSS10, though the difference among ACFC003/12, EUSS11, and SS21 was not significant.

Genotypes varied with environments for cane yield (Table 4). Among the genotypes, SS21 gave the lowest yield across environments during the first season. Since all the genotypes except SS04 showed their highest cane yield in Sagam, the environment of Sagam seems to favor better performance of the genotypes. EUSS10 showed the highest cane in Masumbi, Mundika I, Mundika II, and Sagam, though its cane yield was at medium level in Sinyanya. Thus, it was suggested that EUSS10 was more suited for lower midland zones 1 and 2 whose rainfall and temperatures range between 804 and 846 mm and 20.3 and 29.0°C, respectively. Genotypes that took long to mature grew taller and recorded high cane yield showing a positive relationship between plant height and cane yield. These morphological characters together with stalk diameter and number of internodes per stalk have been reported to affect final yield in sugarcane [22, 23]. Hence, tall sweet sorghum genotypes should be selected to maximize cane yield.

### 3.2. Influence of Environment on Juice Yield

Juice yield differed significantly among the sweet sorghum genotypes; there were high and low performers (Table 5). Among the genotypes, EUSS10 gave the highest juice yields in Masumbi, Mundika I, Mundika II, and Sagam. All genotypes recorded the highest juice yield in Sagam except SS04 and ACFC003/12. Though genotypes performed differently across environments, LM1 agroecological zones (Masumbi and Sagam) favored better performance. In Sinyanya, SS14 recorded about seven times juice yield recorded by SS21. Similarly, EUSS10 recorded juice yield about 3.2 times that recorded by SS21 in Mundika during the first season. Among the genotypes, SS21 and EUSS17 gave relatively low juice yield in Masumbi and Nyahera, respectively. Genotypes responded differently to the varied environments during seasons one and two. The genotypes performed better in Sagam and Masumbi (LM1) due to high total rainfall experienced during the growth period.

### 3.3. Influence of Environment on Brix and Ethanol

Genotypes EUSS11 recorded consistent high Brix values across environments with the controls SS04, SS14, and ACFC003/12 except in Sagam (Table 6). Among the genotypes, EUSS10 had the least total soluble solids (Brix) across environments except in Mundika during season 1. Genotypes recorded similar Brix in Mundika during the 2nd season ranging from 12 to 15.7%. Though SS21 performed poorly in terms of morphological characters, it was the best for Brix in Mundika during seasons I and II and in Sagam. A high Brix value shown by genotypes in Sinyanya is attributed to higher temperatures experienced at that site. John and Seebaluck [24] reported that sugarcane requires higher solar radiation during initial growth stage and during ripening in order to accumulate more sucrose at ripening.

Genotypes varied within environments for ethanol yield (Table 7). Genotypes performed similarly during the 2nd season with SS17, EUSS10, and SS04 recording the lowest volume of ethanol per hectare in Mundika, Nyahera, and Sagam, respectively. In Nyahera, EUSS10 and EUSS17 had ethanol yield that was lower than that produced by other genotypes by about 47–51%. During the season I, ethanol yield of the two controls, SS21 and SS17, was the lowest in Masumbi and Sinyanya and in Mundika, respectively. The maximum ethanol yield among the genotypes across environments was recorded by EUSS11 (838 l/ha) in Sagam. Performance of EUSS17, EUSS10, and EUSS11 was comparable to the best controls SS04, SS14, and ACFC003/12 in most of the tested environments.

The test locations vary in latitude, rainfall, soil types, and temperature. The three environments with high yielding potential, Masumbi, Mundika, and Sagam, are characterized by high bimodal rainfall patterns as compared to lowest yielding environments, Sinyanya and Nyahera. LM1 and LM2 agroecological zones can be utilized for commercial production of sweet sorghum. Genotypes showed satisfactory yields in the most favorable environments (LM1) such as Sagam and Masumbi, the reason being the ability of genotypes to respond advantageously to a higher amount of rainfall in LM1 compared to LM3 agroecological zones. High temperatures and low precipitation are some of contributing factors to poor performance in LM3 AEZ. The slightly better performance of genotypes in Mundika during season one compared to the second season could be due to the difference for rainfall during early growth stages of sorghum plants. Since the fluctuation of ethanol yield of SS14 was smaller than other genotypes, it was suggested that its ethanol yield would be superior to other genotypes in unfavorable environments.

### 3.4. AMMI Stability Values Analysis

The combined analysis of variance of cane and juice yield of sorghum genotypes showed that sweet sorghum genotypes were affected by environments (E), genotypes (G), and genotype by environment interaction (GEI) (Table 8). However, assessment of genotype by environment interaction on ethanol yield stability indicated that GIE was not present for ethanol yield indicating that genotypes did not respond differently to varying environmental conditions. G, E, and GEI effects accounted for 8.6, 36.9, and 19.4%, respectively, for cane yield total sum of squares; 16.8, 24.3, and 22.2%, respectively, for juice yield total sum of squares and 7.1, 38.4, and 18.5%, respectively, for ethanol yield total sum of squares (Table 8). It is important to note that environment contributed largely to variation in yields.

A large sum of squares shows that environments were diverse, influencing yields differently which was in harmony with the findings of Reddy et al. [12] in sweet sorghum production. Traits such as green biomass, plant height, stem diameter, juice extractability, and stem sugar content are major contributors of sweet sorghum's economic importance for biofuel production [25, 26]. However, variability exists in morphological characters of sweet sorghum among genotypes and across locations. Identification of adaptable, stable, and high yielding genotypes under different environmental conditions prior to release has been reported by Lule et al. [14] to be the first and foremost steps for plant breeding. Environment expresses most of the total yield variation while genotype and genotype by environment interactions are less effective [27]. The soil's constituents such as moisture content, mineral availability, and pH that is an integral part of environment cause large annual variation in yield performance of a crop. GEI can be reduced by identifying genotypes that are most stable [28].

The first interaction principal component (IPCA 1) and the second (IPCA 2) accounted for 8.53 and 6.47%, respectively, of the cane's IPCA sum squares. The IPCA1 accounted for 8.22 and 8.26% of juice and ethanol yield interaction sum of squares, respectively, while IPCA2 accounted for 6.97 and 5.07% (Table 8). The first two principal component axes were significant and thus best explain interaction sum of squares and were used in cane and juice yield analysis. However, AMMI model 1 can be used when only one principal component axis is significant to explain the interaction between genotype and environment [21] as for ethanol yield in our case.

Environments and genotypes with least ASV scores are considered as they are the most stable. Accordingly, genotypes SS14, SS17, and ACFC003/12 had a general adaptation for cane yield while SS14 was the most stable for juice and ethanol yield (Table 9). On the other hand, SS04 was most unstable for cane, juice, and ethanol yield. Similarly, environments were classified using ASV as stable for cane yield (Sinyanya, Mundika seasons 1 and II), juice yield (Masumbi, Mundika season I and Sinyanya) and ethanol yield (Mundika season II and Sinyanya). Nyahera and Sagam were the least stable for cane yield while Sagam was unstable for both juice and ethanol yield (Table 10).

Furthermore, the IPCA2 scores of genotypes in AMMI analysis indicate stability of genotypes across locations; high IPCA2 scores (either negative or positive) are unstable while those with low scores are stable [11]. Table 9 showed that genotypes ACFC003/12, SS17, and SS14 for cane yield, SS14, SS04, and EUSS11 for juice yield, and EUSS10, EUSS11, and SS04 for ethanol yield were the most stable genotypes as they had low IPCA2 scores. The most unstable genotypes were SS04 and SS21 for cane yield, SS17 and EUSS10 for juice yield, and SS21, SS17, and EUSS17 for ethanol yield. Stable genotypes follow genes that affect the trait in question and their expression relative to the environment being similar to average cultivar while unstable genotypes have genes that are challenged differently by a different environment [29]. Data in Table 10 further revealed that Masumbi had the highest IPCA2 score for both cane and ethanol while Mundika season II had highest IPCA2 score for juice yield; hence they were the most interactive environments. Sinyanya, Nyahera, and Mundika season II were the least interactive for cane, juice, and ethanol yield, respectively.

### 3.5. GGE Biplot Analysis

**Figure 3:**
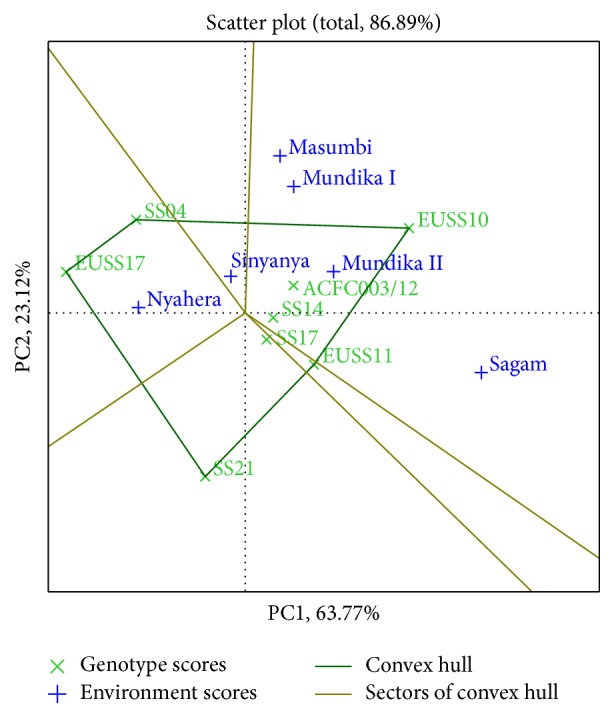
The which-won-where view of GGE biplot for cane yield.

**Figure 4:**
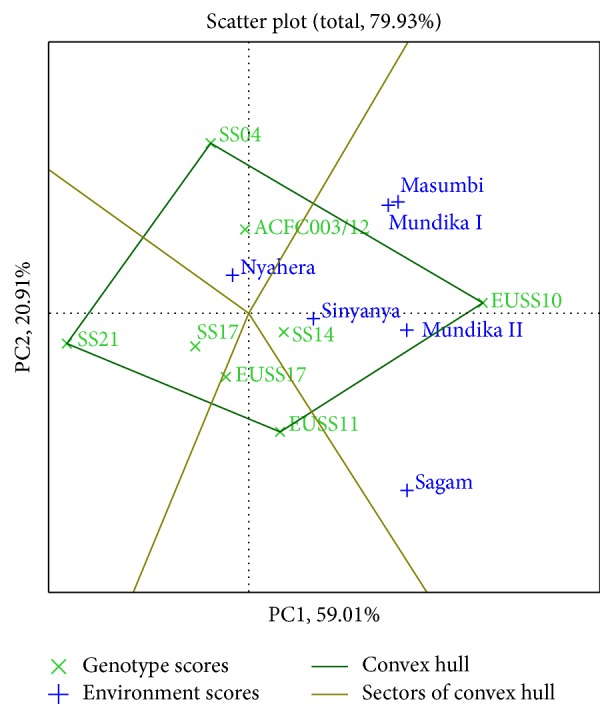
The which-won-where view of GGE biplot for juice yield.

**Figure 5:**
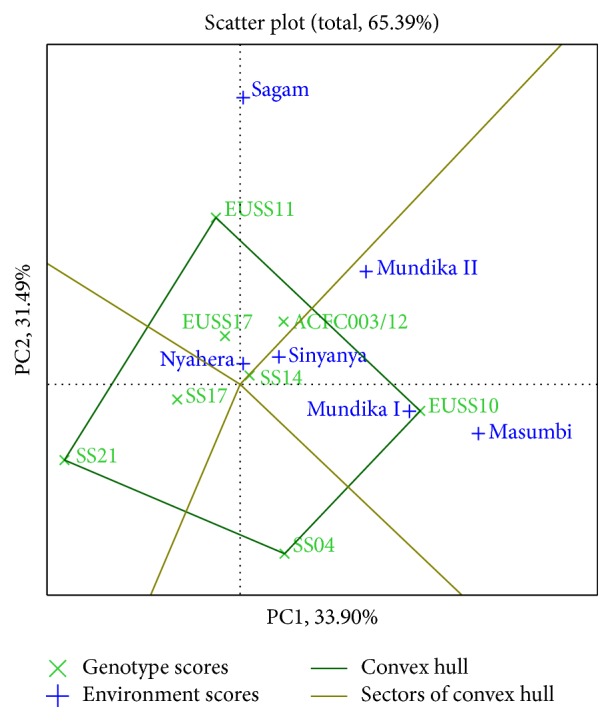
The which-won-where view of GGE biplot for ethanol yield.

Genotypes or environments located on the right-hand side of the midpoint of the axis (IPCA1) have higher yields than those on the left-hand side [29]. In this study, genotypes EUSS10, ACFC003/12, SS14, and EUSS11 for cane yield (Figure 3), EUSS10, EUSS11, and SS14 for juice yield (Figure 4), and EUSS10, SS04, SS14, and ACFC003/12 for ethanol yield (Figure 5) were generally high yielding as they were placed on right-hand side of midpoint of IPC1 axis (representing grand mean). Similarly, Mundika seasons I and II and Sagam and Masumbi were considered to be superior in cane yield (Figure 3), while all sites except Nyahera produced high juice yield (Figure 4). However, all sites performed above average in terms of ethanol yield (Figure 5).

The polygon view of GGE biplot for cane yield (Figure 3) indicates the best genotypes(s) for each environment(s). The genotypes EUSS10, ACFC003/12, and SS14 were found to be promising in Masumbi, Sagam, and Mundika seasons I and II (LM1 and LM2). EUSS17 and SS04 were better adapted to Nyahera (LM3) which is low-performing site. The genotypes located on the vertex of a polygon are the ones that gave the highest yield for the environment that fall within that quadrant. The vertex genotypes were EUSS17, SS04, SS21, EUSS10, and EUSS11 for cane yield. Genotype EUSS10 recorded the highest cane in Masumbi and Mundika during seasons I and II. EUSS11 gave the highest cane in Sagam while both SS04 and EUSS17 were best-performing genotypes in Nyahera and Sinyanya. The polygon reflects that SS21 is poor cane yielding, not suitable for either of the environments. The genotypes located on the vertex of a polygon are best or poorest genotypes in some or all environments except left-bottom quadrant [11].

The GGE biplot for juice yield (Figure 4) indicates that SS14 and EUSS10 are suitable for cultivation in Mundika during seasons I and II, Masumbi, Sinyanya, and Sagam (LM1, LM2, and LM3) while ACFC003/12 and SS04 were better adapted to Nyahera (LM3). EUSS10 recorded the highest juice volume in Sagam, Masumbi, and Mundika during seasons 1 and 2. Genotypes SS21, SS17, and EUSS17 fell into sectors where there were no locations. These genotypes are poorly adapted to all environments that were tested. Locations in one sector having best-performing genotype can be considered as megaenvironments for that genotype [30]. These results are in conformity with the findings of Reddy et al. [12] who observed high yielding and stable genotypes for cane and juice yield.

Biplots were divided into four sectors in Figure 5; genotypes which fall in same sector as with environment are said to be adapted to those locations. In the present study, genotypes EUSS10 and SS14 were adapted to Masumbi, Mundika seasons I and II, and Sinyanya (LM1, LM2, and LM3). EUS11, ACFC003/12, and EUSS17 were suitable for cultivation in Nyahera and Sagam (LM1 and LM3). Furthermore, Figure 5 displays “which-won-where” feature of biplots. EUSS11 had the highest ethanol yield in Sagam. SS21 and SS17 were poor performers for ethanol yield and were not suitable for tested environments.

In Figure 3, genotypes SS14 and SS17 for cane yield had the shortest vector from origin, whereas, in Figures 4 and 5, SS14 for both juice and ethanol yield was closer to the origin than SS17. Moreover, SS14 genotype had IPCA1 > 0 and is therefore regarded as stable and high yielding. Genotype EUSS10 had the highest IPCA1 score and was located close to IPC2 axis for both juice and ethanol yield, indicating that it is high yielding genotype but specifically adapted. Dynamic as opposed to static stability is preferred by breeders and agronomist in order to have genotypes that could produce more yields when optimal agronomic inputs and favorable environmental conditions are provided [31]. Therefore, SS14 can be chosen for wider adaptability and EUSS10 for favorable environments. Genotype ACFC003/12 had medium stability for cane and ethanol yield across environments.

**Figure 6:**
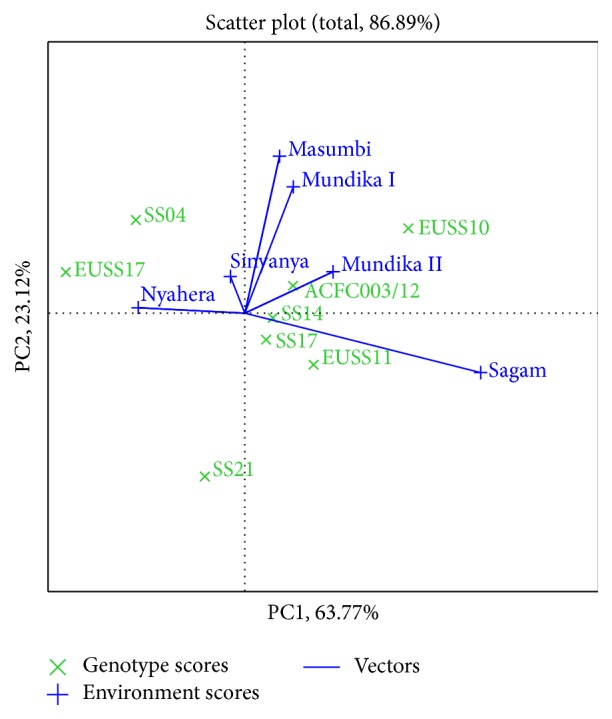
The biplot showing relationship between testers and mega environments for cane yield.

**Figure 7:**
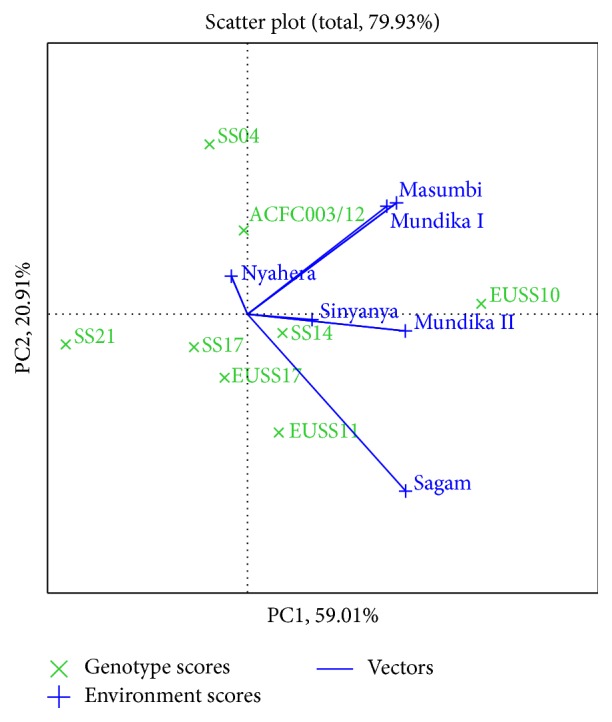
The biplot showing relationship between testers and mega environments for juice yield.

**Figure 8:**
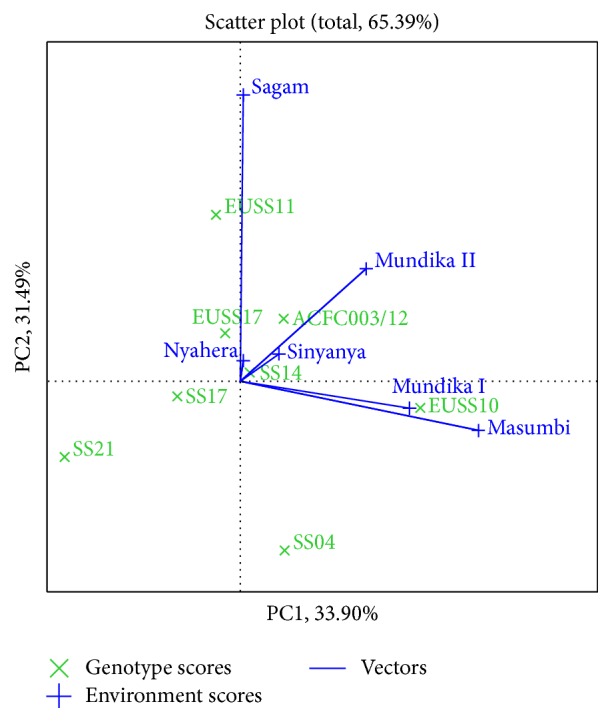
The biplot showing relationship between testers and mega environments for ethanol yield.

Figures 6, 7, and 8 give vector view of GGE biplot of cane, juice, and ethanol, respectively, in which environments are connected with biplot origin via lines. They also show the relationship among genotypes. This view of biplot aids in the understanding of interrelationship among environments. The GGE biplot was applied by Rao et al. [32] to explain the interrelationship among the environments and the seasons. The cosine of the angle between the vectors of two environments approximates the correlation coefficient between them. Environments with a small angle between them are highly positively correlated, and they provide similar information on genotypes. Present investigations showed that Masumbi and Mundika for cane, juice, and ethanol yield (Figures 6, 7, and 8) and Nyahera and Sinyanya for ethanol yield (Figure 8) were considered to be similar as they had small angle between them. In contrast, genotypes EUSS10 and SS21, SS04, and EUSS11 were located in opposing quadrants for cane, juice, and ethanol yields; therefore, the angles between them were larger and are considered as dissimilar genotypes. Similarly, Nyahera and Sagam were dissimilar for both cane and juice yield. Sinyanya and Nyahera lied closest to the origin and, therefore, contributed the least to GEI for cane, juice, and ethanol yield while Sagam made the highest contribution. From this study, it is evident that low-performing genotypes are stable and have wider adaptability, whereas high-performing genotypes are less stable.

A study by Abubakar and Bubuche [33] in Nigeria found out that genotype by environment interaction had a significant influence on sorghum plant height. Differences in plant height can result in changes in cane yield across environments; therefore, genotypes adapted to specific locations have to be selected. Biomass yield and plant height have been found to be major contributors to economic yields in sweet sorghum [34]. Furthermore, ANOVA revealed there was a significant effect due to genotype by environment interaction. This indicates that genotypes performed differently at each site, which is expected due to differences in soil composition, rainfall, and temperature. Ideal cultivars and environments are those having large PC1 scores (high mean yield) and small PC2 scores (high stability) [35]. Based on this, Mundika season I and Masumbi were found to be ideal environments whereas SS14 was ideal genotype for ethanol production. Genotype EUSS10 was the winning genotype for ethanol yield in Masumbi and Mundika both in seasons one and two and in Sinyanya and, therefore, suitable for those sites.

## 4. Conclusion and Recommendations

Cane yield was found to be highly correlated with plant height. Environmental effects, as well as GEI, had strong effect on yield of sweet sorghum genotypes. The significant GEI for cane and juice yield observed from analysis of variance in this study shows that sweet sorghum genotypes respond differently when grown in different environmental condition. The results from this project indicate that SS14 was most stable and best genotype across environments whereas EUSS10 has excellent potential for ethanol in areas with high yield potential. The best-performing genotypes were EUSS10, ACFC003/12, and SS04 while average performers were EUSS11, EUSS17, and SS14. The genotypes SS21 and SS17 were poor performers for ethanol yield located outside limits of any environments. It is evident that performance of sweet sorghum is attributed to both genetic make-up and environment.

## Figures and Tables

**Table 1:** Description of soil components of testing locations.

County	site	^*∗∗*^AEZ	pH	Soil type	Nitrogen (%)	Phosphorous (ppm)
Siaya	Masumbi	^*∗*^LM1	4.4	Clay loam	0.11	9.8
Siaya	Sinyanya	LM3	5.4	Sandy clay loam	0.17	8.8
Busia	Mundika I	LM2	4.4	Sandy clay loam	0.09	6.4
Busia	Mundika II	LM2	4.4	Sandy clay loam	0.09	6.4
Kisumu	Sagam	LM1	5.8	Sandy clay loam	0.12	8.5
Kisumu	Nyahera	LM3	6.0	Sandy clay loam	0.15	5.5

^*∗*^Lower midland zone. ^*∗∗*^Agroecological zone.

**Table 2:** Influence of environment on maturity (days to 50% heading) of sweet sorghum genotypes.

Genotypes	Environments					
	Masumbi (LM1)	Mundika (LM2)	Sinyanya (LM3)	Mundika (LM2)	Nyahera (LM3)	Sagam (LM1)
	Number of days to 50% heading					
	Season 1 (March–July)			Season 2 (Sept–Dec)		
SS04	79.33^abc^	80.00^b^	80.33^ab^	69.33^bcd^	73.00^ab^	76.33^abcd^
SS14	87.00^a^	85.00^a^	85.00^ab^	76.00^a^	74.00^ab^	82.00^ab^
SS21	73.00^c^	71.67^c^	66.00^c^	66.33^d^	61.33^c^	66.00^d^
SS17	76.33^bc^	79.33^b^	80.33^ab^	67.30^cd^	71.33^b^	72.67^bcd^
EUSS17	79.67^abc^	80.33^b^	78.00^b^	72.33^abc^	75.33^ab^	78.33^abc^
EUSS10	84.33^ab^	86.33^a^	86.33^a^	74.00^ab^	76.67^a^	85.00^a^
EUSS11	80.33^abc^	81.33^b^	80.33^ab^	72.67^abc^	75.00^ab^	77.00^abc^
ACFC003/12	80.00^abc^	79.00^b^	82.00^ab^	70.67^abcd^	73.00^ab^	70.00^cd^
LSD_0.05_	8.45	2.81	7.99	5.61	4.23	10.54

Means followed by the same letter do not differ at the same column at 5% LSD.

**Table 3:** Plant height of sweet sorghum genotypes across environments.

Genotypes	Environments					
	Masumbi (LM1)	Mundika (LM2)	Sinyanya (LM3)	Mundika (LM2)	Nyahera (LM3)	Sagam (LM1)
	Plant height (cm)					
	Season 1 (March–July)			Season 2 (Sept–Dec)		
SS04	181.17^ab^	215.17^ab^	107.33^a^	203.44^abc^	175.22^ab^	197.67^a^
SS14	176.67^ab^	194.17^c^	111.0^a^	209.44^ab^	162.44^b^	217.33^a^
SS21	140.33^c^	199.33^c^	85.33^a^	178.56^bc^	175.67^ab^	195.00^a^
SS17	190.33^ab^	143.50^d^	108.00^a^	175.22^c^	173.78^ab^	218.00^a^
EUSS17	184.50^ab^	205.67^bc^	108.00^a^	196.11^abc^	166.78^b^	208.33^a^
EUSS10	206.33^a^	226.67^a^	105.67^a^	219.89^a^	181.44^ab^	232.67^a^
EUSS11	167.00^bc^	191.33^c^	91.00^a^	182.11^bc^	167.56^b^	223.33^a^
ACFC003/12	172.83^abc^	204.67^bc^	81.67^a^	205.00^abc^	189.44^a^	210.67^a^
LSD_0.05_	36.27	18.40	34.49	32.01	21.32	40.39

Means followed by the same letter do not differ at the same column at 5% LSD.

**Table 4:** Cane yield (t/ha) of eight sweet sorghum genotypes across environments.

Genotypes	Environments					
	Masumbi (LM1)	Mundika (LM2)	Sinyanya (LM3)	Mundika (LM2)	Nyahera (LM3)	Sagam (LM1)
	Cane yield (t/ha)					
	Season 1 (March–July)			Season 2 (Sept–Dec)		
SS04	26.39^ab^	28.47^ab^	13.89^ab^	15.97^b^	22.22^a^	19.44^b^
SS14	24.31^ab^	20.82^cd^	16.69^a^	20.83^b^	16.65^ab^	32.64^ab^
SS21	9.72^b^	13.20^e^	8.33^b^	16.72^b^	20.14^a^	28.47^ab^
SS17	27.78^ab^	15.28^de^	13.89^ab^	16.56^b^	15.97^ab^	33.33^ab^
EUSS17	25.00^ab^	18.06^cde^	16.67^a^	16.63^b^	10.15^b^	33.32^ab^
EUSS10	31.94^a^	30.81^a^	12.50^ab^	29.97^a^	11.81^b^	44.44^a^
EUSS11	18.75^ab^	22.92^bc^	13.89^ab^	23.61^ab^	21.53^a^	40.28^a^
ACFC003/12	23.61^ab^	24.31^bc^	11.11^ab^	25.69^ab^	21.53^a^	34.72^ab^
LSD_0.05_	19.02	7.63	6.04	10.73	7.66	17.54

Means followed by the same letter do not differ at the same column at 5% LSD.

**Table 5:** Juice yield (l/ha) of eight sweet sorghum genotypes across environments.

Genotypes	Environments					
	Masumbi (LM1)	Mundika (LM2)	Sinyanya (LM3)	Mundika (LM2)	Nyahera (LM3)	Sagam (LM1)
	Juice yield (l/ha)					
	Season 1 (March–July)			Season 2 (Sept–Dec)		
SS04	8044^ab^	6304^bc^	3567^ab^	4225^bc^	4518^a^	4011^c^
SS14	7014^ab^	5090^cd^	5061^a^	5588^bc^	3649^ab^	8611^abc^
SS21	1938^b^	2850^e^	761^c^	4281^bc^	4000^ab^	4763^c^
SS17	7086^ab^	3472^de^	3310^ab^	2188^c^	3311^ab^	8442^abc^
EUSS17	7057^ab^	3907^de^	4617^ab^	4063^bc^	2332^b^	8135^abc^
EUSS10	9615^a^	9051^a^	3861^ab^	10111^a^	3056^ab^	11146^a^
EUSS11	4867^ab^	4849^cde^	3411^ab^	6344^b^	4646^a^	10647^ab^
ACFC003/12	5990^ab^	7364^ab^	2406^bc^	5674^bc^	5028^a^	6269^bc^
LSD_0.05_	6346	2141	2307	3587	1975	4731

Means followed by the same letter do not differ at the same column at 5% LSD.

**Table 6:** Brix (%) of sweet sorghum genotypes across environments.

Genotypes	Environments					
	Masumbi (LM1)	Mundika (LM2)	Sinyanya (LM3)	Mundika (LM2)	Nyahera (LM3)	Sagam (LM1)
	Brix (%)					
	Season 1 (March–July)			Season 2 (Sept–Dec)		
SS04	18.3^a^	18.0^a^	19.0^a^	15.0^a^	16.7^ab^	13.7^abc^
SS14	16.7^ab^	14.8^abc^	21.0^a^	13.0^a^	17.0^ab^	13.3^abc^
SS21	14.0^cd^	18.3^a^	15.7^bc^	15.7^a^	12.3^c^	17.0^a^
SS17	16.3^b^	11.7^c^	18.3^ab^	15.0^a^	15.3^b^	15.3^ab^
EUSS17	15.7^bc^	13.0^c^	18.7^ab^	15.0^a^	15.0^b^	15.0^ab^
EUSS10	12.3^d^	13.3^bc^	15.0^c^	12.0^a^	9.3^d^	10.7^c^
EUSS11	17.0^ab^	15.3^abc^	18.7^ab^	15.0^a^	17.7^a^	13.0^bc^
ACFC003/12	17.0^ab^	14.8^abc^	18.3^ab^	15.3^a^	16.3^ab^	17.0^a^
LSD_0.05_	1.9	4.7	3.0	4.1	2.3	3.9

Means followed by the same letter do not differ at the same column at 5% LSD.

**Table 7:** Ethanol yield (l/ha) of sweet sorghum genotypes across environments.

Genotypes	Environments					
	Masumbi (LM1)	Mundika (LM2)	Sinyanya (LM3)	Mundika (LM2)	Nyahera (LM3)	Sagam (LM1)
	Ethanol yield (l/ha)					
	Season 1 (March–July)			Season 2 (Sept–Dec)		
SS04	539.0^ab^	247.3^bc^	276.2^abc^	358.6^ab^	350.2^a^	325.4^b^
SS14	469.9^ab^	170.1^c^	371.1^a^	402.0^ab^	212.3^ab^	629.1^ab^
SS21	129.8^c^	129.7^c^	117.0^d^	351.7^ab^	199.8^ab^	450.9^ab^
SS17	474.8^ab^	112.8^c^	174.4^cd^	204.8^b^	194.0^ab^	676.6^ab^
EUSS17	472.8^ab^	119.8^c^	317.4^ab^	369.3^ab^	147.5^b^	722.2^ab^
EUSS10	644.2^a^	417.3^a^	177.4^cd^	568.5^a^	137.8^b^	573.0^ab^
EUSS11	326.1^ab^	154.9^c^	244.5^abcd^	574.3^a^	337.3^ab^	838.1^a^
ACFC003/12	401.3^ab^	336.3^ab^	224.4^bcd^	500.3^a^	377.2^a^	698.7^ab^
LSD_0.05_	425.2	152.21	138.4	282.7	229.36	429.78

Means followed by the same letter do not differ at the same column at 5% LSD.

**Table 8:** Additive main effects and multiplicative interaction analysis of variance for cane, juice, and ethanol yield of the genotypes across environments.

Source of variation	DF	Cane yield			Juice yield			Ethanol yield		
		SS	MS	Explained (%)	SS	MS	Explained (%)	SS	MS	Explained (%)
Total	143	2100813	14691	—	1306609520	9137130	—	7724869	54020	—
Treatments	47	446829	9507^*∗∗∗*^	64.71	827867592	17614204	63.36	4944624	105205^*∗∗∗*^	64.01
Genotypes	7	8799	1257^*∗∗*^	8.56	220070892	31438699^*∗∗∗*^	16.84	548971	78424^*∗*^	7.11
Environments	5	27115	5423^*∗∗∗*^	36.91	317801649	63560330^*∗∗∗*^	24.32	2963587	592717^*∗∗∗*^	38.36
Block	12	11484	957	—	70966844	5913904	—	313774	26148	—
Interaction	35	98910	2826^*∗*^	19.44	289995052	8285573^*∗*^	22.19	1432066	40916^ns^	18.54
IPCA1	11	13783	1253^*∗*^	8.53	107425316	9765938^*∗*^	8.22	638384	58035^*∗*^	8.26
IPCA2	9	8550	950^*∗*^	6.47	91016710	10112968^*∗*^	6.97	391863	43540^ns^	5.07
Residuals	15	9345	623	—	91553026	6103535	—	401819	26788	—
Error	84	4225	50.3	—	407775083	4854465	—	2466471	29363	—

^*∗*^ *P* < 0.05; ^*∗∗*^ *P* < 0.01; ^*∗∗∗*^ *P* < 0.001; ns: nonsignificant; DF: degrees of freedom; SS: sum of square; MS: mean square.

**Table 9:** The first two IPCA scores and ASV for genotypes.

Genotype	Cane yield				Juice yield				Ethanol yield			
	Mean (t/ha)	PCA1	PCA2	ASV	Mean (l/ha)	PCA1	PCA2	ASV	Mean (l/ha)	PCA1	PCA2	ASV
SS04	21.06	−3.10	2.07	4.58	5112	−50.24	−18.57	62.14	349.5	−13.18	3.76	21.80
SS14	21.99	−0.06	0.32	0.33	5835	11.11	−13.30	18.68	375.8	0.19	−4.64	4.65
SS21	16.10	−1.17	−2.56	2.99	3099	−17.65	27.03	34.12	229.8	2.97	10.12	11.22
SS17	20.47	0.30	0.70	0.80	4635	14.65	−38.73	42.43	306.2	1.74	−9.28	9.70
EUSS17	20.01	0.87	1.07	1.57	5018	17.68	−29.10	35.81	358.2	3.82	−9.02	10.96
EUSS10	26.91	2.90	1.02	3.96	7807	25.14	34.07	45.17	419.7	−10.19	−1.04	16.64
EUSS11	23.50	0.39	−1.94	2.01	5794	30.20	18.18	40.01	412.5	12.31	3.69	20.40
ACFC003/12	2350	−0.13	−0.68	0.17	5455	−30.88	20.44	41.79	423.1	2.34	6.41	7.46

**Table 10:** AMMI stability values of cane, juice, and ethanol yield for 8 sweet sorghum genotypes evaluated in different environments.

Environments	Cane yield				Juice yield				Ethanol yield			
	Mean (t/ha)	PCA1	PCA2	ASV	Mean (l/ha)	PCA1	PCA2	ASV	Mean (l/ha)	PCA1	PCA2	ASV
Masumbi	23.44	0.49	2.96	3.03	6451	−6.83	−36.33	37.21	432.2	−11.81	−11.14	22.24
Mundika I	21.73	−0.58	1.19	1.41	5361	−26.19	22.27	38.09	211.0	−7.78	5.53	13.83
Mundika II	20.75	0.96	−0.98	1.60	5309	6.01	48.53	49.04	416.2	1.66	1.66	3.18
Nyahera	17.53	−3.04	−1.87	4.42	3817	−36.46	0.45	43.04	244.5	1.54	8.64	8.99
Sagam	33.33	3.00	−1.72	4.32	7753	62.33	1.63	73.58	614.2	16.00	−7.11	27.02
Sinyanya	13.37	−0.84	0.42	1.18	3374	1.14	−36.53	36.56	237.8	0.39	−4.01	4.06
